# Evidence for separate backward recall and *n*-back working memory factors: a large-scale latent variable analysis

**DOI:** 10.1080/09658211.2024.2393388

**Published:** 2024-08-26

**Authors:** Elizabeth M. Byrne, Rebecca A. Gilbert, Rogier A. Kievit, Joni Holmes

**Affiliations:** a School of Psychology, University of East Anglia, Norwich, UK; b MRC Cognition & Brain Sciences Unit, University of Cambridge, Cambridge, UK; c Department of Brain & Cognitive Sciences, Massachusetts Institute of Technology, Cambridge, MA, USA; d Donders Institute, Radboud University, Nijmegen, Netherlands

**Keywords:** Working memory, Backward recall, *n*-back, Fluid intelligence, Reasoning, Latent variable analysis

## Abstract

Multiple studies have explored the factor structure of working memory (WM) tasks, yet few have done so controlling for both the domain and category of the memory items in a single study. In the current pre-registered study, we conducted a large-scale latent variable analysis using variant forms of n-back and backward recall tasks to test whether they measured a single underlying construct, or were distinguished by stimuli-, domain-, or paradigm-specific factors. Exploratory analyses investigated how the resulting WM factor(s) were linked to fluid intelligence. Participants (*N* = 703) completed a fluid reasoning test and multiple n-back and backward recall tasks containing memoranda that varied across (spatial or verbal material) and within (verbal digits or letters) domain, allowing the variance specific to task content and paradigm to be assessed. Two distinct but related backward recall and n-back constructs best captured the data, in comparison to other plausible model constructions (single WM factor, two-factor domain, and three-factor materials models). Common variance associated with WM was a stronger predictor of fluid reasoning than a residual n-back factor, but the backward recall factor predicted fluid reasoning as strongly as the common WM factor. These data emphasise the distinctiveness between backward recall and n-back tasks.

Working memory (WM) supports a wide range of complex behaviours, including reading comprehension, following instructions, and problem-solving (Feldman Barrett et al., 2004; Holmes et al., 2021; Jaroslawska et al., 2018; Peng & Kievit, 2019). WM varies between individuals and can be measured using a variety of paradigms (e.g., backward recall, complex span or n-back). While multiple studies have explored the shared variance between different WM paradigms (e.g., Kane et al., 2007; Redick & Lindsey, 2013; Schmiedek et al., 2009; Schmiedek et al., 2014), sometimes controlling for the domain (verbal or visuo-spatial) of the memory items (e.g., Kovacs et al., 2019), few have done so controlling for content modality (differences in the domain *and* category of the memory items) across tasks. Waris et al. (2017) tested whether WM tasks could be distinguished by process (e.g., updating or maintenance) or “content”, but content was defined as either numerical-verbal or visuo-spatial, and did not distinguish between different materials within domain (e.g., digits or letters). The aim of the current study was to conduct a large-scale latent variable analysis controlling for both the domain and category of the memoranda using variant forms of n-back and backward recall tasks to test whether they measure a single underlying construct, or are distinguished by stimuli-, domain-, or paradigm-specific factors.

Many WM paradigms combine the temporary storage of information with additional processing requirements such as reversing a sequence (backward recall), updating the contents of WM (n-back), or handling interpolated distractor tasks (complex span), although measures capturing the maintenance of bindings between items in WM, without explicit processing, are equally suited to measure WM capacity (Oberauer, 2019; Wilhelm et al., 2013). There are verbal (digits, letters) and visuo-spatial (spatial locations) variants of each of these paradigms, and in the case of complex span tasks the distractor items can also vary by domain. Multiple latent variable analyses have examined the construct validity of WM tasks, and individual differences studies have explored how WM tasks predict other complex cognitive tasks such as fluid reasoning (Alloway et al., 2006; Chuderski, 2013; Engle, Laughlin, et al., 1999; Kane et al., 2007; Oberauer et al., 2000; Schmiedek et al., 2009; Shamosh et al., 2008).

Complex span tasks containing different memory items and distractor activities correlate extremely well with each other and with other measures of WM, including updating tasks such as n-back (e.g., Schmiedek et al., 2009). They also predict performance on tests of language comprehension (Daneman & Carpenter, 1980; Kane et al., 2004), attentional control (Kane et al., 2008), and general fluid reasoning (G*f*; e.g., Schmiedek et al., 2009). Associations between different forms of n-back and other WM paradigms are weaker (Dobbs & Rule, 1989; Jaeggi, Buschkuehl, et al., 2010; Jaeggi, Studer-Luethi, et al., 2010; Kane et al., 2007; McAuley & White, 2011; Miller et al., 2009; Redick & Lindsey, 2013; Roberts, 1998; Roberts & Gibson, 2002), and n-back has been used less often to predict other cognitive abilities (Kane et al., 2007).

Few studies have validated backward recall tasks against other tests of WM, and those that have done so typically focus on backward digit recall (BDR; e.g., Hilbert et al., 2015). This task relies on short-term memory serial order mechanisms to maintain digit sequences. The additional requirement to recall items in reverse order imposes a substantial attentionally-demanding processing load similar to the executive loads of other WM tasks (Alloway et al., 2006; Bull et al., 2008). Evidence from a meta-analysis that backward span is more strongly related to n-back than to simple forward span tasks (Redick & Lindsey, 2013), and that it is associated with reasoning ability (e.g., Suß et al., 2002), supports the argument that backward recall has an executive component (although see Colom et al., 2005; Engle, Laughlin, et al., 1999; St Clair-Thompson & Allen, 2013 for arguments that BDR is a short-term memory task). Indeed, Redick and Lindsey’s meta-analysis (2013) reported that the correlation between n-back and backward digit span (*r *= .31) was greater than the correlation between n-back and verbal complex span (*r *= .18), suggesting not only that it shares variance with other widely used WM tasks, but also that it may have more in common with some WM paradigms than others.

There are a number of methodological issues that limit the conclusions that can be drawn from the majority of previous studies exploring shared variance between WM tasks (Schmiedek et al., 2009; Schmiedek et al., 2014; Wilhelm et al., 2013). First, task associations could be reduced due to a mismatch of content modality across paradigms (e.g., differences in the domain or category of the memory items). For example, weak correlations between n-back and complex span reported by Kane et al. (2007) could reflect differences in task stimuli (n-back contained letters, complex span combined word recall with numerical operations), rather than differences in paradigm.

Using a single indicator for each paradigm can also be problematic because variants of the same paradigm can provide different indices of an underlying factor. For example, Kane et al. (2004) reported that different complex span tasks (operation, reading, counting, navigation, rotation, or symmetry span) explained different amounts of variance in a single underlying WM construct. Using a single measure for any paradigm therefore introduces task-specific variance into latent models (Shipstead et al., 2012). When performance is averaged across multiple versions of a WM task, stronger associations are found between constructs (Schmiedek et al., 2014). For example, Shamosh et al. (2008) reported a higher correlation between latent factors of two n-back tasks and four complex span tasks than Kane et al. (2007) who measured associated measures of performance on single n-back and complex span tasks.

In the present study we use a latent variable analysis to test whether backward recall and n-back measures of WM tap into the same underlying construct, or whether the tasks are distinguished by stimuli-, domain-, or paradigm-specific factors. The two paradigms have been reported to be weakly associated in previous studies (Dobbs & Rule, 1989; McAuley & White, 2011; Miller et al., 2009; Roberts, 1998; Roberts & Gibson, 2002; and for a meta-analysis, see Redick & Lindsey, 2013), but these studies have been limited by the shortcomings of using single task indicators and not controlling for the influence of domain– and task-specific variance. To address these issues, we included multiple indicators of WM that vary the overlap in task properties by WM paradigm (backward recall, n-back), stimulus domain (verbal, visuo-spatial), and stimulus material (digits, letters). Confirmatory factor analysis was used to test four competing models of the underlying structure of the tasks.

The first candidate model tested was a single-factor WM model where all backward recall and n-back tasks loaded on one factor (see Figure 1A). This is consistent with domain-general theories of WM proposing that performance on WM tasks is dependent on a domain-general central executive or attentional control system (Alloway et al., 2006; Baddeley, 1986; Engle & Kane, 2004; Engle, Kane, et al., 1999; Kane et al., 2004). The second candidate model was a two-factor, domain-specific model encompassing distinct but related verbal and visuo-spatial factors (shown in Figure 1B). This aligns with models proposing that separate pools of resources support the maintenance and processing of verbal and visuo-spatial information (Daneman & Tardif, 1987; Friedman & Miyake, 2000; Shah & Miyake, 1996). The third candidate model tested was a two-factor paradigm model (e.g., Schmiedek et al., 2009). Both backward recall and n-back tasks require the temporary maintenance and processing of information, and require the recollection of previously presented information (e.g., backward recall requires explicit serial recall, while n-back involves recollecting whether the current item has been presented n + 1, n + 2 or more steps back) (Oberauer, 2005). However, familiarity-based retrieval might introduce additional noise in n-back tasks, distinguishing the two paradigms. For this reason, the two-factor paradigm model assumes a correlation between two distinct backward recall and n-back latent constructs. This candidate model is shown in Figure 1C. The final model tested was a three-factor materials model that assumed performance across the tasks would be best captured by expertise related to the specific type of stimuli (e.g., basic skills or knowledge tied to digits, letters, or spatial materials). This three-factor model assumed separate constructs for each category of memory item as follows: (i) n-back with digits and backward recall with digits; (ii) n-back with letters and backward letter recall; and (iii) n-back with spatial locations and backward spatial recall. This model is shown in Figure 1D. The protocol for this part of the study was preregistered on the Open Science Framework (https://osf.io/9qarp/).

**Figure 1.**
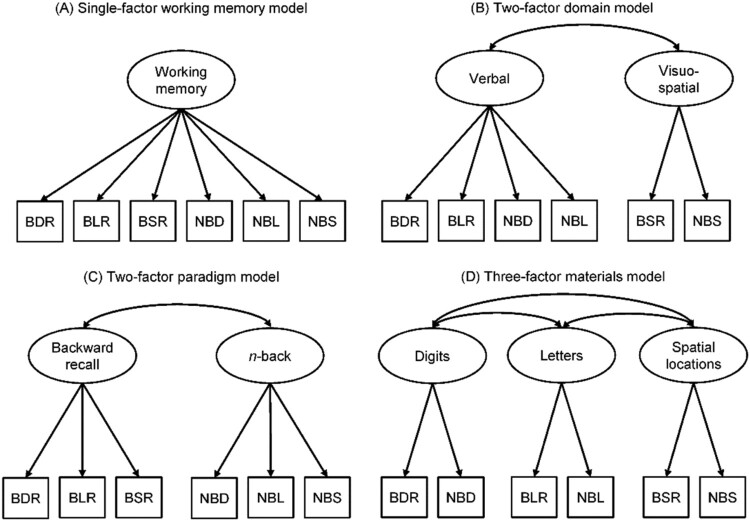
Candidate models for the primary analyses. Models A (single-factor working memory), B (two-factor domain), C (two-factor paradigm), and D (three-factor materials). Ovals represent latent factors and observed variables are shown in squares. BDR = backward digit recall, BLR = backward letter recall, BSR = backward spatial recall, NBD = n-back with digits, NBL =* *n-back with letters, NBS = n-back with spatial locations.

In a second pre-registered stage, we explored the association between the best fitting WM factor model from Figure 1 and fluid reasoning. High correlations between WM and fluid reasoning (e.g., Conway et al., 2002; Kane et al., 2005) have led some to argue that they are isomorphic constructs (e.g., Duncan et al., 2000; Kyllonen & Christal, 1990). Others, however, argue that they are distinct but related constructs (Ackerman et al., 2005; Chuderski, 2013; Conway et al., 2003; Fukuda et al., 2010; Kane et al., 2004; Oberauer et al., 2005; Schmiedek et al., 2009, 2014). It is unclear what processes might support the relationship between WM and fluid intelligence, and the current study was not designed to tease among these, but it is worth noting that this topic is widely debated. Some argue WM and fluid reasoning are highly related as they both rely on the ability to control attention (Engle, 2018; Kane et al., 2007; Shipstead et al., 2016), while others suggest that the WM processes of building, maintaining, and manipulating arbitrary bindings between items supports performance on fluid reasoning tasks (Oberauer et al., 2007). A final proposal suggests that the relationship between the two is best explained by similar demands on short-term memory storage (Colom et al., 2006 ; Colom et al., 2008).

The aims of our exploratory analyses were to test whether a single factor model (with all WM and reasoning tasks loading on one factor) provided a better account of the data than a model with separate but related reasoning and WM factors. In a final set of analyses, which were exploratory and not pre-registered, we decided that if the model with separate but correlated WM and reasoning factors was the better fit than the single factor model, we would explore whether there were differences in the strength of the associations between the different WM factors and fluid reasoning. We planned these analyses to test whether we could replicate previous studies suggesting that different WM tasks might make differential contributions to fluid reasoning (e.g., Shipstead et al., 2012). We also explored whether the variance common to all WM tasks was a stronger predictor of fluid reasoning than the variance unique to either the backward recall or n-back paradigms. The domain-general view of WM would predict that the common variance among WM task variants should predict reasoning more strongly than the variance unique to any paradigm (e.g., Kane et al., 2004).

## Supplementary Material

Supplemental Material

## Figures and Tables

**Figure 2.**
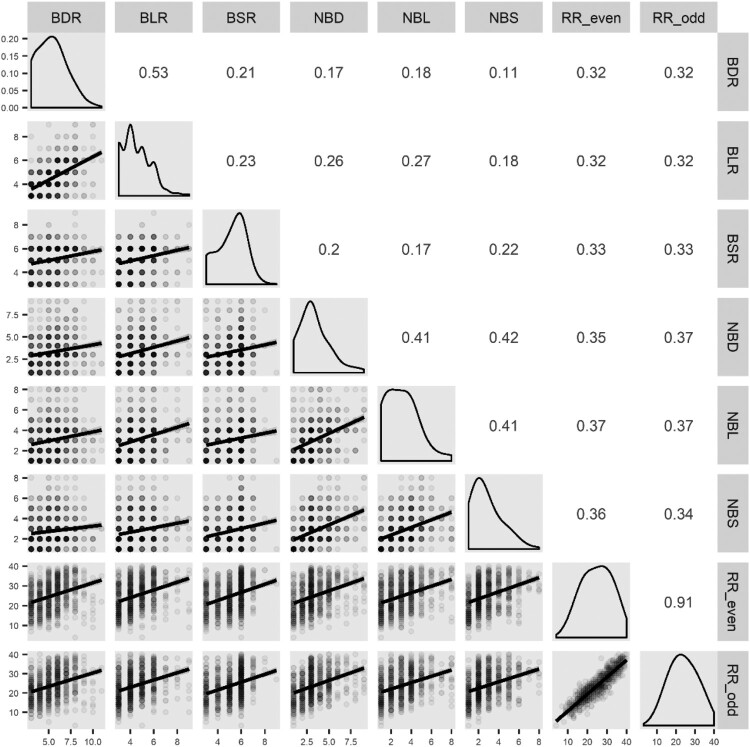
Matrix of associations between pairs of tasks (*N *= 703). Simple correlation coefficients are displayed in the top segment (all significant at *p *< .01), density plots are shown along the diagonal, and scatter plots with trend lines are displayed in the lower section. BDR = backward digit recall, BLR = backward letter recall, BSR = backward spatial recall, NBD = n-back with digits, NBL = n-back with letters, NBS = n-back with spatial locations, RR_even = relational reasoning with even items, and RR_odd = relational reasoning with odd items.

**Table 1.** Descriptive statistics for all variables.

Variable	N	M	SD		Skewness	Kurtosis
Backward digit recall	698	5.307	1.695		.479	−.187
Backward letter recall	690	4.470	1.247		.844	.704
Backward spatial recall	702	5.068	1.214		−.405	−.734
n-back digits	694	3.307	1.723		.977	1.047
n-back letters	698	3.032	1.658		.757	.380
n-back spatial locations	699	2.774	1.511		.984	.591
Relational reasoning even	700	24.921	7.604		−.096	−.879
Relational reasoning odd	700	23.787	7.431		.037	−.683

**Table 2.** Fit statistics for each model included in the primary confirmatory analysis.

Model		χ²	df	YB	RMSEA	CFI	AIC
(A)	Single-factor WM	195.825	9	.932	.172 [.151, .194]	.678	14739
(B)	Two-factor domain	204.926	8	.889	.187 [.164, .211]	.660	14741
(C)	Two-factor paradigm	29.108	8	.977	.061 [.038, .086]	.964	14587
(D)	Three-factor materials	189.847	6	.826	.209 [.181, .237]	.683	14720

Note. For root mean errors of approximation (RMSEAs), 90% confidence intervals are given. CFI = comparative fit index; AIC = Akaike information criterion. The χ² reported is the Yuan-Bentler scaled χ², with the scaling factor reported as YB. WM = working memory.

**Table 3.** Fit statistics for each model included in the primary confirmatory analysis with residual covariance between the two verbal backward recall tasks.

Model		χ^2^	df	YB	RMSEA	CFI	AIC
(E)	Single-factor WM with BDR & BLR residual covariance	24.154	8	.996	.054 [.030, .079]	.972	14583
(F)	Two-factor domain with BDR & BLR residual covariance	25.036	7	.953	.061 [.035, .088]	.969	14585
(G)	Two-factor paradigm with BDR & BLR residual covariance	**10**.**658**	7	.970	.027 [.000, .059]	.994	14571

Note. **Bold** text denotes a non-significant χ² value. For root mean errors of approximation (RMSEAs), 90% confidence intervals are given. CFI = comparative fit index; AIC = Akaike information criterion. The χ² reported is the Yuan Bentler scaled χ², with the scaling factor reported as YB. WM = working memory, BDR = backward digit recall, BLR = backward letter recall.

**Figure 3.**
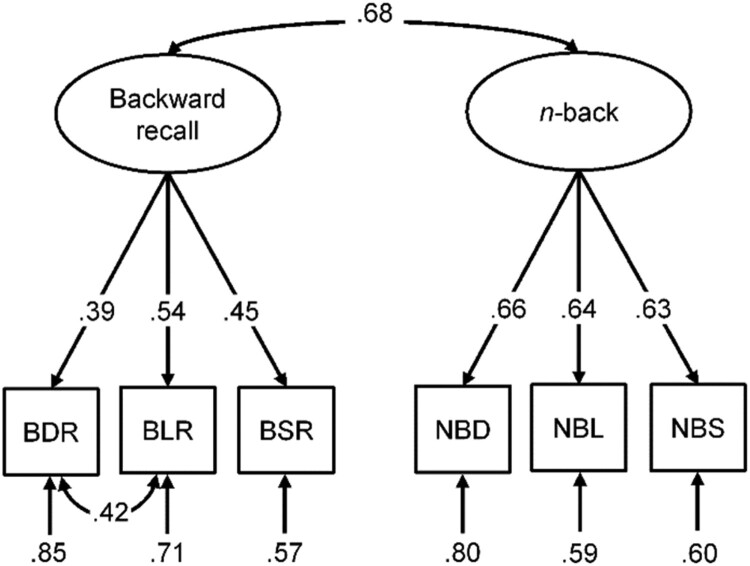
The best model from the primary analyses (Model G); a two-factor paradigm-based model with residual covariance between the two verbal backward recall tasks. Latent factors are shown in ovals and squares represent observed variables. Confidence interval for the latent correlation between backward recall and n-back, 95% [.533, .823], based on Maximum Likelihood (ML) bootstraps. BDR = backward digit recall, BLR = backward letter recall, BSR = backward spatial recall, NBD = n-back with digits, NBL = n-back with letters, NBS = n-back with spatial locations. All parameter estimates shown are fully standardised. Note that a version of this model without the residual covariance between the two verbal backward recall tasks (Model C described above) is shown in the Supplementary Materials (Figure S1) for comparison. Adding residual covariance between the two verbal backward recall tasks strengthens the relationship between the two latent constructs and causes the loadings of BLR and BDR on to the backward recall factor to decrease and the loading of the BSR task on to this factor to increase. These changes reflect the fact that Model G takes into account the common variance (correlated error term) between BLR and BDR; that is, the phonological encoding and retrieval processes in verbal short-term memory that are common to both tasks.

**Figure 4.**
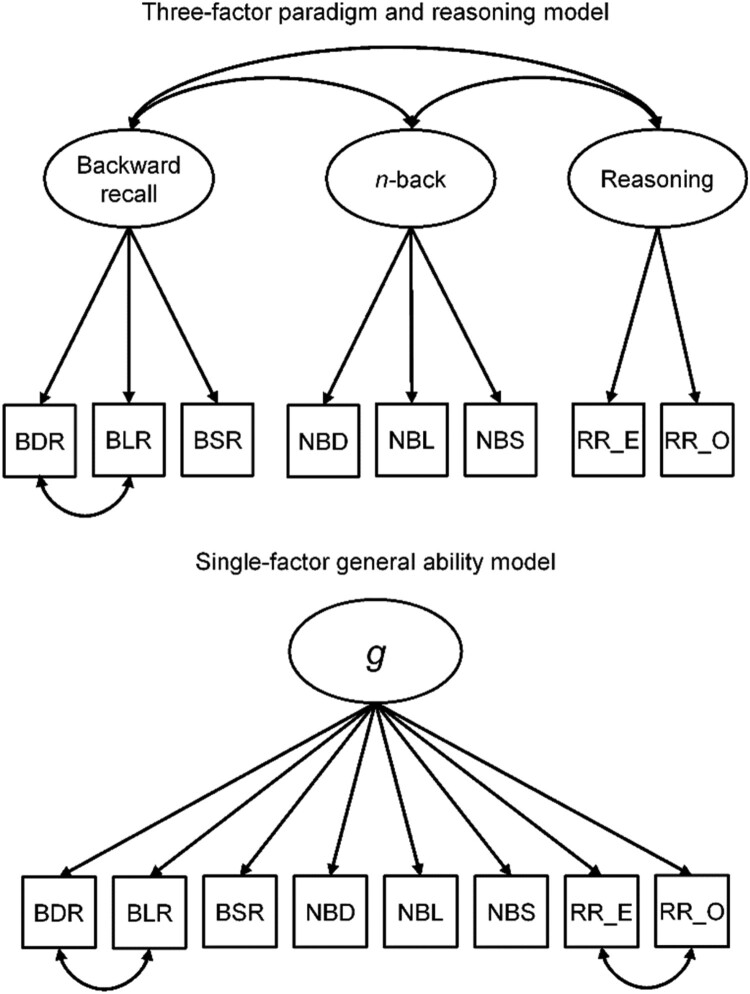
Candidate models for the exploratory analyses. The top panel shows the three-factor paradigm and reasoning model (H), and the bottom panel displays the single-factor general ability model (I). Ovals represent latent factors and observed variables are shown in squares. BDR = backward digit recall, BLR = backward letter recall, BSR = backward spatial recall, NBD = n-back with digits, NBL =* *n-back with letters, NBS = n-back with spatial locations, RR_E = relational reasoning even items, RR_O = relational reasoning odd items. Note that the residual covariance between the two verbal backward recall tasks is retained in these models, but models without this are tested for completeness (Models J and K) and reported in the Supplementary Materials (Figures S2 and S3, and Table S1).

**Table 4.** Fit statistics for the models included in the exploratory analyses.

Model		χ²	df	YB	RMSEA	CFI	AIC
(H)	Three-factor paradigm & reasoning with BDR & BLR residual covariance	**23**.**576**	16	1.006	.026 [.000, .047]	.996	22752
(I)	Single-factor general ability with BDR & BLR residual covariance, and RR_E & RR_O residual covariance	65.683	18	1.009	.061 [.046, .078]	.975	22791

Note. **Bold** text denotes a non-significant χ² value. For root mean errors of approximation (RMSEAs), 90% confidence intervals are given. CFI = comparative fit index; AIC = Akaike information criterion. The χ² reported is the Yuan Bentler scaled χ², with the scaling factor reported as YB. RR_E = relational reasoning even items, RR_O = relational reasoning odd items, BDR = backward digit recall, BLR = backward letter recall. The fit statistics for models without the residual covariance between BDR and BLR are summarised in the Supplementary Materials (Table S1).

**Figure 5.**
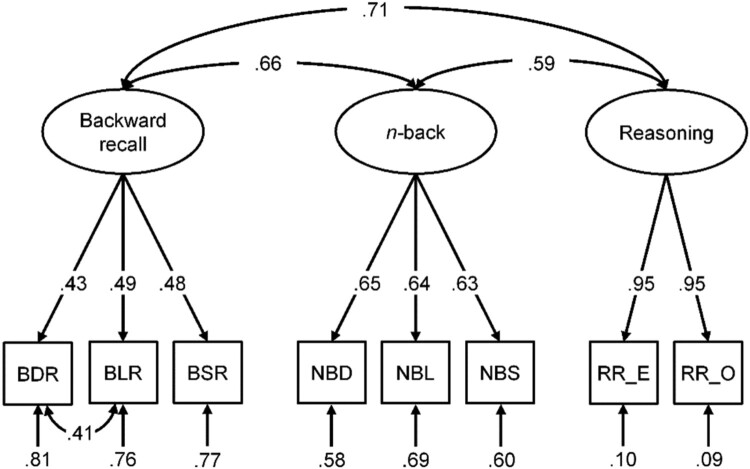
The winning model from the exploratory analyses (Model H); a three-factor paradigm and reasoning model with residual covariance between the two verbal backward recall tasks. Latent factors are shown in ovals and observed variables are represented by squares. BDR = backward digit recall, BLR = backward letter recall, BSR = backward spatial recall, NBD = n-back with digits, NBL = n-back with letters, NBS = n-back with spatial locations, RR_E = relational reasoning even items, RR_O = relational reasoning odd items. All parameter estimates shown are fully standardised. Note that a version of this model without the residual covariance between the two verbal backward recall tasks (Model J) is shown in the Supplementary Materials (Figure S2) for comparison. Including the residual covariance between the two verbal tasks did not affect the association between the n-back and Reasoning factors, but the association between the backward recall factor and both other factors increased.

**Figure 6.**
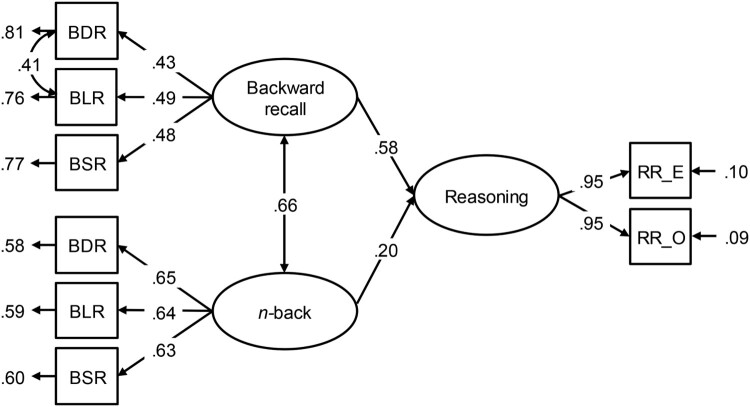
SEM model from the exploratory analyses (Model L) with residual covariance between the two verbal backward recall tasks. Latent factors are shown in ovals and observed variables are represented by squares. BDR = backward digit recall, BLR = backward letter recall, BSR = backward spatial recall, NBD = n-back with digits, NBL = n-back with letters, NBS = n-back with spatial locations, RR_E = relational reasoning even items, RR_O = relational reasoning odd items. All parameter estimates shown are fully standardised and freely estimated. This model was compared to an equality constrained model where the paths between both WM factors and reasoning were assumed to be equal (fit statistics for both models are provided in Supplementary Materials, Table S2).

**Figure 7.**
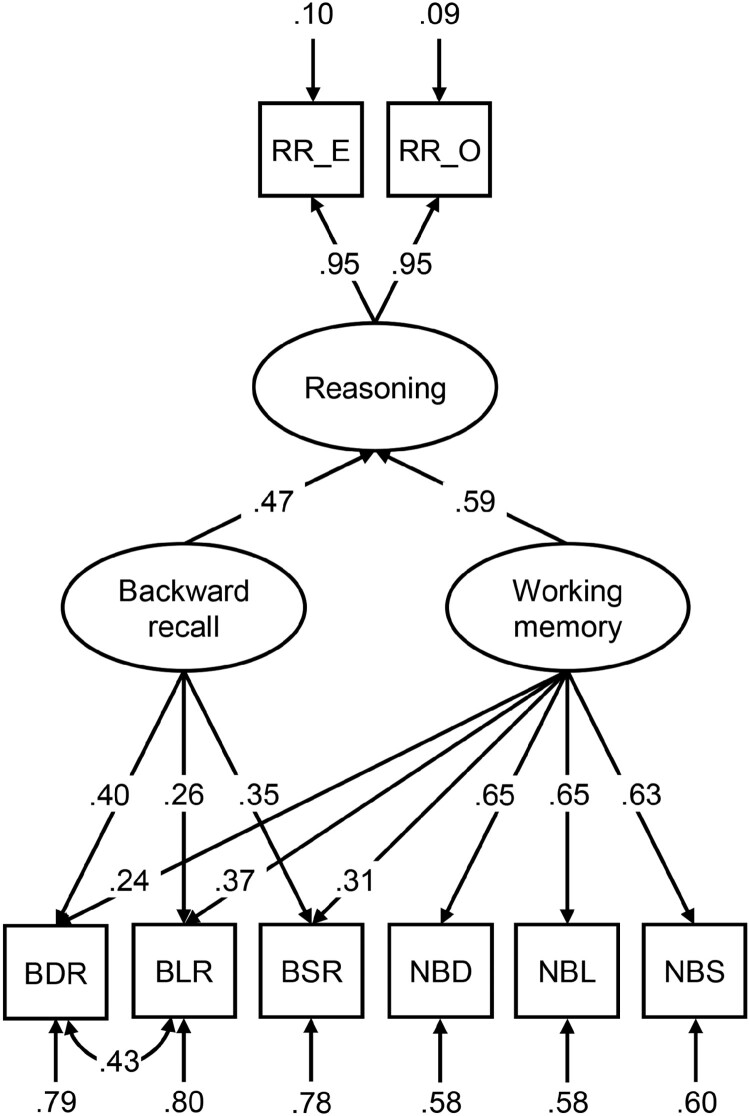
Bifactor model (Model N) modelling the prediction of reasoning by a common WM variance factor and a residual backward recall factor, with residual covariance between the two verbal backward recall tasks. Latent factors are shown in ovals and observed variables are represented by squares. BDR = backward digit recall, BLR = backward letter recall, BSR = backward spatial recall, NBD = n-back with digits, NBL = n-back with letters, NBS = n-back with spatial locations, RR_E = relational reasoning even items, RR_O = relational reasoning odd items. All parameter estimates shown are fully standardised and freely estimated. This model was compared to an equality constrained model where the paths between both WM factors and reasoning were assumed to be equal (fit statistics for both models are provided in Supplementary Materials, Table S4).

**Figure 8.**
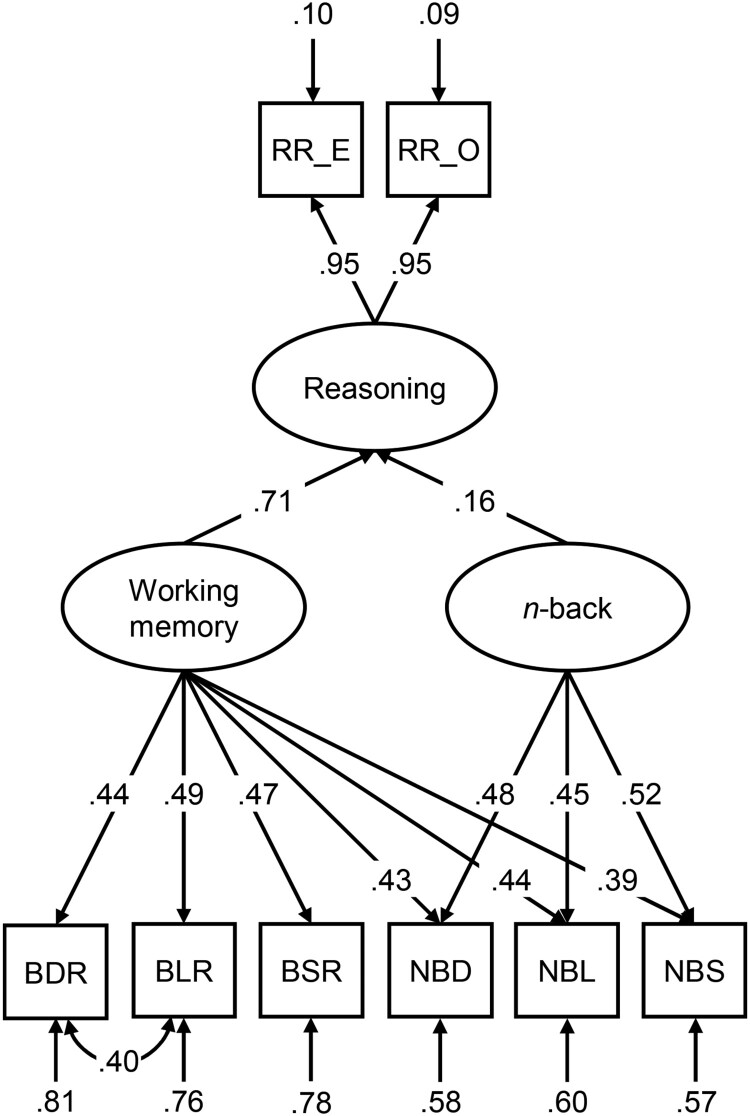
Bifactor model (Model O) modelling the prediction of reasoning by a common WM variance factor and a residual n-back factor, with residual covariance between the two verbal backward recall tasks. Latent factors are shown in ovals and observed variables are represented by squares. BDR = backward digit recall, BLR = backward letter recall, BSR = backward spatial recall, NBD = n-back with digits, NBL = n-back with letters, NBS = n-back with spatial locations, RR_E = relational reasoning even items, RR_O = relational reasoning odd items. All parameter estimates shown are fully standardised and freely estimated. This model was compared to an equality constrained model where the paths between both WM factors and reasoning were assumed to be equal (fit statistics for both models are provided in Supplementary Materials, Table S5).

## Data Availability

The data and R analysis script for this study have been made openly available via the Open Science Framework online repository for this project, which can be accessed here: https://osf.io/9qarp/.

## Method

### Participants

Seven-hundred and seven native-English speaking participants aged 18-35 completed this study and were paid £9 for participation. All had normal or corrected to normal vision, and no literacy difficulties. Data was excluded for four participants who did not follow the study instructions correctly. A total sample size of 703 participants (421 female) was used for the analyses (mean age = 27.476, SD = 4.474). Participants were recruited through Prolific Academic (https://www.prolific.com/; Palan & Schitter, 2018; Peer et al., 2017) and completed the tasks online. Only 29 out of over 5000 observations were identified as extreme outliers, and there were only 14 cases of missing data due technical problems. To foreshadow the results, the quality of the data enabled us to detect cognitive factor structure(s) underlying nuanced individual differences that are consistent with those reported in lab-based studies (e.g., Chuderski, 2013; Engle, Laughlin, et al., 1999; Schmiedek et al., 2009, 2014). Consistent with other studies, these data show high quality cognitive data can be collected via web-browser platforms and produced outputs comparable to data collected in the lab (Crump et al., 2013).

Informed consent was obtained online prior to testing. The study was approved by and conducted in accordance with the guidelines of the University of Cambridge Psychology Research Ethics Committee (Reference: PRE.2017.001).

### Procedure

Each participant completed six memory tasks and a fluid reasoning task in a single session according to one of 12 possible task orders. The backward recall tasks were grouped together (i.e., completed consecutively), and the n-back tasks were also grouped together. The task order within these two groups was counterbalanced (i.e., all possible permutations for the three tasks were used), yielding six orders for each of the two groups of tasks. The order of the two groups of backward recall and n-back tasks was then counterbalanced, resulting in six possible task orders in which the backward recall tasks were completed first, and six in which the n-back tasks were completed first. An additional reasoning task was completed in between the n-back and backward recall tasks in all conditions (i.e., it was always the fourth task completed). Participants completed practice trials before starting each task. Feedback for correct and incorrect responses was shown on screen for the practice trials but was not provided during the proper tasks.

### Materials

The tasks were created using the software programme Gorilla (https://gorilla.sc/; Anwyl-Irvine et al., 2018). Participants completed the study on a laptop or desktop computer, and all responses were made using a mouse or keyboard.

#### Backward recall

Participants completed three backward recall tasks each containing different stimuli: (i) digits (1–9), (ii) letters (B H J L N Q R X Z), or (iii) spatial locations (nine random but fixed locations on the computer screen). Trials were presented in blocks, with each block consisting of four trials. During each trial, items were presented visually one at a time (stimulus presentation = 750 ms, inter-stimulus interval = 250 ms). Participants were then prompted to recall the sequence in-backward order via an onscreen keypad of digits, letters, or spatial locations. Participants began each task at a span of three items. Span length was increased by one item in each subsequent block if there were three or more correct responses out of the four trials at that length. The tasks were discontinued if two or more trials were incorrect within a block, or if the highest possible span level was reached (13 items). For each of the backward recall tasks, we scored participants according to their maximum span (i.e., the final span length in which the participant met the criterion of at least three out of four correct trials).

#### n-back

Participants completed three n-back tasks, each containing different stimuli: (i) digits (1–9), (ii) letters (B H J L N Q R X Z), or (iii) spatial locations. For each task, stimuli were presented randomly, one at a time on screen in a random order (stimulus presentation = 760 ms, inter-stimulus interval = 2000 ms), with no deliberate placement of lures (although some will have occurred by chance). Participants were required to indicate whether the current item on screen matched the one presented *n* items back in the sequence via a button press. In each block participants were presented with a continuous sequence of 20 + *n* items, during which there were a total of six possible targets (matches) and 14 + *n* non-targets. An error was scored if participants pressed the button for a non-target (false alarm), or if participants failed to press the button when a match was present (miss). Total errors were calculated as false alarms plus misses. The first block began at one-back and difficulty level was increased by one in each subsequent block if fewer than five errors were made (e.g., an increase from one-back to two-back). The task ended if five or more errors were made within a block, or if the highest possible level was reached (12-back). We scored each of the n-back tasks according to the maximum n-level that the participant reached (i.e., the final level in which the participant met the criterion of less than five errors in a block).

#### Relational reasoning

Participants were presented with 80 puzzles one at a time (Knoll et al., 2016). The stimuli were developed from a set of materials that have recently been normed (Fuhrmann et al., 2018). Although the stimuli used here are not identical to those normed by Fuhrmann et al. (2018), they were developed from the same source. Each puzzle consisted of a 3 × 3 matrix (nine spaces in total). Eight of the spaces contained shapes, and the bottom right space was always empty. Participants were also presented with four boxes at the bottom of the screen containing shapes, and were required to select the box with the correct answer – the box containing the piece that was missing from the empty space in the matrix. The shapes in the matrix varied by colour, size, shape, and position. Difficulty level also varied. Participants were given 30 s to complete each trial, and a prompt appeared on screen when only 5s remained. Odd and even items were scored separately to generate two relational reasoning scores (so that a latent reasoning factor could be formed using the two measures for the exploratory analyses). In each case the number of correct responses (out of 40) was used.

### Analysis plan

To address the primary pre-registered research question, we ran a series of confirmatory factor analyses to determine which of the four candidate models (see Figure 1) best explain the covariance structure among the six WM tasks. The following models were compared: (A) a single WM factor model, (B) a two-factor domain-specific verbal and visuo-spatial construct model, (C) a two-factor backward recall and n-back paradigm model, and (D) a three-factor digit, letter, and spatial materials model.

After establishing the best-fitting WM model for the variables, a set of exploratory analyses that were not pre-registered were conducted to test how the two classes of WM measures were related to fluid reasoning. The configuration of the best-fitting WM model was retained, and a reasoning factor was added to explore whether the WM factor(s) and the reasoning tasks load on a single factor, or on distinct but related constructs. If a single-factor WM model was preferred, the plan was to examine whether the WM factor is very strongly or perfectly correlated with a fluid reasoning factor. Alternatively, if a multi-factor model was preferred, then the relationship between the WM factors and fluid reasoning would be examined to see whether the relationship was stronger for different WM sub-factors. The multi-factor models were compared to a single-factor general ability model including all WM and reasoning tasks.

### Model fit and comparison

Models were estimated in the *lavaan* software package (version 0.6-14; Rosseel, 2012) in R version 4.1.3 (R Core Team, 2018) using maximum likelihood estimation and robust standard errors, for which the Yuan-Bentler (YB) scaled test statistic is reported. Missing observations were dealt with using the full maximum likelihood (FIML) parameter estimation technique because it allowed us to maximise the utility of all existing data and increase power relative to deleting incomplete cases (Baraldi & Enders, 2010). The overall fit of each model was assessed using the χ² test, the comparative fit index (CFI; range: 0-1.0; acceptable fit: .95-.97, good fit: ≥ .97; Schermelleh-Engel et al., 2003), and the root mean square error of approximation (RMSEA; range: 0-1.0; acceptable fit: < .08, good fit: ≤ .05; Schermelleh-Engel et al., 2003) which is reported with 90% confidence intervals. The four models were also compared. When models were nested, they were compared via a likelihood ratio test (i.e., the Satorra-Bentler scaled χ² difference test; Satorra & Bentler, 2001); otherwise non-nested models were directly compared via the Akaike information criteria (AIC; Akaike, 1974).

## Results

### Preliminary analyses

The data were screened to identify outliers (i.e., scores deviating > 3.5 SDs from the sample mean on each task). Twenty-nine observations were removed during data screening for outliers, and an additional 14 observations were missing due to technical problems during data collection (total missing observations = 37). Descriptive statistics are summarised in Table 1. Associations between tasks are displayed in Figure 2. All tasks were positively correlated (all *p*s < .01). The strongest patterns of association were observed between-backward digit and backward letter recall (*r* = .526), and between the three n-back tasks (all *r*s > .4). The two relational reasoning scores were very highly correlated (*r* = .91), suggesting they are reliable indicators of the construct.

### Primary analyses

#### WM factor models

Confirmatory factor analysis was used to compare the preregistered measurement models of the six memory tasks. The models tested are displayed in Figure 1. Fit indices for each model are provided in Table 2. The fit statistics revealed that the single-factor model (Figure 1A), χ^2^ (9) = 195.825, RMSEA = 0.172 (90% confidence interval [CI] = .151, .194), and CFI = .678, the two-factor domain model (Figure 1B), χ^2^ (8) = 204.926, RMSEA = .187 (90% CI = .164, .211), and CFI = .660, and the three-factor materials model (Figure 1D), χ^2^ (6) = 189.847, RMSEA = .209 (90% CI = .181, .237), and CFI = 683, were a poor fit to data. In contrast, the two-factor paradigm model (Figure 1C), χ^2^ (8) = 29.108, RMSEA = .061 (90% CI = .038, .086), and CFI = .964, was an acceptable fit to the data.

The fit of the single-factor WM model (Figure 1A) was compared with each of the other models using the Yuan-Bentler χ^2^ difference tests because it was nested within the other models. These analyses revealed that the fit of the single-factor model (A) was not significantly different to the domain model (B), Δ χ^2^ = .150, Δ *df* = 1, *p* = .700, but it did provide a significantly better account of the data than the materials model (D), Δ χ^2^ = 22.367, Δ *df* = 3, *p* < .001. The two-factor paradigm model (C) outperformed the single-factor model (A), Δ χ^2^ = 272.820, Δ *df* = 1, *p* < .001. The AIC measurement was used to directly compare the other models to one another. The two-factor paradigm model (C) was the best fit with the lowest AIC value (see Table 2), suggesting that separate but related latent constructs corresponding to backward recall and n-back best capture the covariance across the tasks (see Figure S1 in the Supplementary Materials for the Model C diagram).

### Improving WM model fit

To explore whether we could improve the fit of the multi-factor models, we inspected the modification indices (MI) and the standardised residual covariance (SRC) estimates. These converged on the same key path for the two-factor paradigm model (*MI* = 17.976, *SRC* = 3.894) and two-factor domain model (*MI* = 149.144, *SRC* = 3.804), namely a strong residual correlation between the BDR and BLR tasks. On reflection, this residual correlation is to be expected due to both tasks depending on encoding and retrieval processes from verbal short-term memory (Norris et al., 2019). As this system stores verbal material in phonological rather than semantic form (e.g., Salamé & Baddeley, 1982), the processes involved in encoding, maintaining, and retrieving representations from verbal short-term memory should be the same for letters as digits. Based on this reasoning, we allow this additional residual covariance path for all models going forward, but report the fit for all models with and without it. This additional parameter was not added to the three-factor materials model, as unlike the within factor cross-loadings in the other models, the inclusion of a between factor cross-loading would weaken the separation between the three individual factors that are central to demarcating this model as distinct from the others. As can be seen in Table 3 the addition of the residual covariance between BDR and BLR improved the fit of all models approximately equally, thus not substantively affecting the core goal of model comparison. The addition of this residual covariance to each model does not have any effect on the preregistered model comparisons.

The revised single-factor WM model (Model E) was a good fit to the data, χ^2^ (8) = 24.154, RMSEA = .054 (90% CI = .030, .079), and CFI = .972. The revised domain model (Model F) also improved and was an acceptable fit to the data, χ^2^ (7) = 25.036, RMSEA = .061 (90% CI = .035, .088), and CFI = .969. A χ^2^ difference test revealed these two models were not significantly different to each other, Δ χ^2^ = .145, Δ *df* = 1, *p* <���.704. The modified two-factor paradigm model (Model G) showed excellent fit to the data, χ^2^ (7) = 10.658, RMSEA = .027 (90% CI = .000, .059), and CFI = .994. The χ^2^ statistic for Model G was non-significant (*p *= .145), a further indication this model was a good fit. A χ^2^ difference test demonstrated that the two-factor paradigm model with the residual covariance between BDR and BLR (Model G) outperformed the single-factor model with the same residual covariance between the verbal backward recall tasks (Model E), Δ χ^2^ = 11.668, Δ *df* = 1, *p* < .001. A likelihood ratio test was not appropriate to compare the revised domain model (F) to the modified paradigm model (G) because these two models (F and G) are not nested, but the AIC values (see Table 3) suggested that model G fit was considerably better than model F (Δ AIC = 14), suggesting that the best-fitting model of the WM tasks overall was the paradigm-based model with residual covariance between the two verbal backward recall tasks (G). This model (Model G) is displayed in Figure 3.

### Exploratory analyses

#### WM and fluid reasoning

Exploratory analyses, listed in the pre-registration, were conducted to explore whether WM and fluid reasoning were isomorphic or separable. We tested whether a model with a separate reasoning factor linked to the two paradigm factors model (Model H) provided a better account of the data defined by a model with all WM and reasoning tasks loading on a single factor (Model I). See Figure 4 for a schematic of these candidate models.

For simplicity, and because the residual covariance between the BLR and BDR tasks is both theoretically defensible and similarly beneficial for all models reported in the primary analysis, we report outcomes using the best-fitting WM model *with* the residual covariance between BDR and BLR (Model G, best-fitting WM-only model) when testing the candidate models linking WM to fluid reasoning (outlined in Figure 4). For completeness the candidate models shown in Figure 4 were also tested using the best-fitting WM model *without* the residual covariance between BDR and BLR (Model C of the WM-only models). A summary of these results and the model comparisons are reported in the Supplementary Materials (see Figures S2 and S3 for models J and K, respectively; see Table S1 for model fit statistics).

The fit statistics for the two models displayed in Figure 4 with the residual covariance between BDR and BLR are shown in Table 4. The first candidate model was comprised of three correlated latent variables – one for each set of backward recall, n-back, and reasoning tasks (Model H; see Figure 4, top panel). The fit indices of this model were excellent: χ^2^ (16) = 23.576, *p *= .099, RMSEA = .026 (90% CI = .000, .047), and CFI = .996.

The second candidate model assumed a single latent construct for all measures (three backward recall, three n-back, and two reasoning tasks; Model I, see Figure 4, bottom panel). As well as the residual covariance between BDR and BLR, the residual covariance between the two reasoning tasks was added, as these are two halves of the same task. This model was an acceptable fit to the data, χ^2^ (18) = 65.683, RMSEA = .061 (90% CI = .046, .078), and CFI = .975 (see Supplementary Materials, Figure S4 for Model I with factor loadings).

A χ^2^ difference test demonstrated that Model H outperformed Model I, Δ χ^2^ = 41.136, Δ *df* = 2, *p* < .001. This was confirmed by the AIC values (see Table 4), revealing that the best-fitting model of the WM and reasoning tasks was a three-factor model with latent constructs corresponding to backward recall, n-back, and fluid reasoning, including the residual covariance between BDR and BLR. The winning model is displayed along with factor loadings in Figure 5. These distinct latent constructs were strongly related to each other, and the relationships between-backward recall and reasoning, and between n-back and reasoning, were similar (.71 and .59, respectively). Individual task loadings on each paradigm factor were similar indicating no single task contributed substantially more variance than another.

To test whether fluid reasoning is separable from WM, additional exploratory analyses were conducted to test whether the residual variance of the fluid reasoning factor was significant after accounting for that predicted jointly by the two WM latent factors. The two paths from the individual WM factors jointly predicted 40.8% of the variance in the latent fluid reasoning factor. The remaining 59.2% of variance was significant (residual variance estimate = 30.836, SE = 2.371, *p* < .001, standardised residual variance = .592). This was confirmed by comparing the AIC of a model where the residual variance was freely estimated to a model which constrained the residual variance of the fluid reasoning to be zero (Δ AIC = 153.2).

#### Do backward recall and n-back differentially predict fluid reasoning?

The winning model (Model H) shows there is a stronger relationship between-backward recall and fluid reasoning (.71) than between n-back and fluid reasoning (.59). In further exploratory analyses, which were not pregistered, we tested whether the two WM factors differentially *predicted* fluid reasoning by converting Model H into a freely varying structural equation model (Model L, see Figure 6). This showed numerically that backward recall was a stronger predictor of fluid reasoning (.58) than n-back (.20). To test whether these differences were significantly different we compared the fit of this model (Model L), in which the paths were freely estimated between each WM factor and reasoning, to a model in which the links between each WM factor and reasoning were constrained to be equal (constrained model). If the backward recall and n-back factors predict reasoning differentially, the freely estimated models will provide a better fit to the data than the constrained models, but if there is no significant difference between the models, the data can be explained equally as well by assuming the links between each WM factor and reasoning are equal. The fit statistics for both models are reported in Table S2. A χ^2^ difference test demonstrated that there was no significant difference between the fit of the two models, Δ χ^2^ = 3.710, Δ *df* = 1, *p* = .054. Repeating these analyses without the residual covariance between the BLR and BDR tasks (Model M) revealed the same pattern: there was no significant difference between the free and constrained models, Δ χ^2^ = 2.899, Δ *df* = 1, *p* = .089 (see Table S3 for fit statistics). These analyses reveal that there is no significant difference in the ways in which backward recall and n-back predict fluid reasoning.

#### Does the common WM variance among tasks predict fluid reasoning more strongly than the variance unique to either the backward recall or n-back paradigms?

Domain-general views of WM predict that the common variance among WM task variants should predict reasoning more strongly than “residual” variance unique to either the backward recall or n-back factors. We tested this through a series of bifactor models. Bifactor models allow for a single factor to be modelled to capture the variance common to all indicators (e.g., a WM factor capturing variance common to all tasks). This provides a partial account of the correlations between the indicators (WM tasks) and allows for specific factors to be modelled to account for the residual correlations between indicators (e.g., variance specific to each WM paradigm). Thus, bifactor models allowed us to model the variance common to all the WM tasks (WM factor), and separate factors to account for the residual correlations between the tasks that was specific to each WM paradigm (residual backward recall and residual n-back factors).

Bifactor models can be conceptually challenging when they are used to make inferences about underlying dimensions and relations between these dimensions and other dependent variables. Criticisms include interpretational problems related to: (i) allowing indicators to be bidimensional (loading on both a common variance and specific residual factor), (ii) allowing common variance factors to explain correlations between indicators when their explanatory value is, arguably, in accounting for correlations between specific (lower-order) factors, and (iii) not allowing for common variance factors to mediate links between specific factors and other outcomes (see Dolan & Borsboom, 2023 for more details). Here we use bifactor modelling as a pragmatic tool to test our specific question about whether the variance common to all WM tasks predicted reasoning more strongly than paradigm-specific variance. We do not use it to comment on the ontology of underlying factors.

We used a series of bifactor models to test whether the common variance among WM task variants (WM factor) predicted reasoning more strongly than residual variance unique to either the backward recall or n-back factors. In the first (Model N, Figure 7) the variance common to all WM tasks (the three backward recall and three n-back tasks, referred to as the “common WM variance”) and a residual backward recall factor were modelled as predictors of the reasoning factor. To explore whether the common WM factor predicted reasoning more strongly than the backward recall factor, the fit of this model (Model N), in which the paths were freely estimated between each WM factor and reasoning, was compared to a model in which the links between each WM factor and reasoning were constrained to be equal (constrained model). Standardisation was used to set the scaling of the factors in the constrained model to ensure the variances of the factors were equal, and thus the correlations between each WM factor and reasoning were equal. The fit statistics for the freely estimated and constrained models are provided in Table S4. A χ^2^ difference test demonstrated that there was no significant difference between the fit of the two models, Δ χ^2^ = 1.415, Δ *df* = 1, *p* = .234, indicating the common WM variance and backward recall factors are equally strong complementary predictors of reasoning, inconsistent with the predictions of a domain-general view of WM.

To explore whether the common WM variance factor predicted reasoning more strongly than the n-back factor both were modelled as predictors of the reasoning factor (Model O, Figure 8). The freely estimated model was compared to a model in which the links between each WM factor and reasoning were constrained to be equal (constrained model; see Table S5 for the fit statistics for both models). Standardisation was used to set the scaling of the factors for the constrained model to ensure the variances of the factors were equal, and thus the correlations between each WM factor and reasoning were equal. A χ^2^ difference test demonstrated that there was a significant difference between the fit of the two models, Δ χ^2^ = 23.419, Δ *df* = 1, *p* = <.001, indicating that the common WM variance and n-back factors differentially predicted reasoning. The freely estimated model (see Figure 8) revealed that the WM common factor explained most variance in reasoning (.71), with a modest contribution of n-back (.16). This is consistent with the domain-general view of WM that predicts a common WM factor should account for more variance in reasoning than anything paradigm specific.

## Discussion

This is the first large-scale latent variable analysis testing the overlap in task features between-backward recall and n-back tasks controlling for content– and material-specific variance. The data were best-captured by two distinct but related (*r* = .68) paradigm constructs for backward recall and n-back. This result is consistent with previous work reporting a paradigm-specific latent structure for other WM tasks (e.g., complex span and updating or n-back paradigms; Schmiedek et al., 2014; Wilhelm et al., 2013). In these previous studies, high latent correlations were reported between paradigm factors represented by variant forms of each task.

The zero-order correlations between individual tasks were relatively small, consistent with those reported in a previous meta-analysis (e.g., Redick & Lindsey, 2013), but the association between the n-back and backward recall latent variables was higher here than in previous studies and meta-analyses (Dobbs & Rule, 1989; McAuley & White, 2011; Miller et al., 2009; Redick & Lindsey, 2013; Roberts, 1998; Roberts & Gibson, 2002). This difference is likely explained by task-specific variance in estimates of performance within paradigms, which lowered correlations in earlier studies (Kane et al., 2004; Schmiedek et al., 2014). A few studies included variant forms of n-back with different verbal and spatial materials (McAuley & White, 2011; Redick & Lindsey, 2013), but the majority used only a single indicator of n-back (Dobbs & Rule, 1989; Miller et al., 2009; Roberts, 1998; Roberts & Gibson, 2002), and all included only one backward recall task (BDR). Using a latent variable approach with multiple indicators of each paradigm, we have overcome the problem that correlations between single tasks are attenuated by paradigm-specific and content-specific sources of individual variation and measurement error (Schmiedek et al., 2009, 2014).

The strong correlation between the two WM factors – the common source of variance between the backward recall and n-back constructs – could reflect commonalities across the tasks, for example in the mechanisms used for building, maintaining, and updating arbitrary bindings between memory items and their serial position (Chatham et al., 2011; Oberauer et al., 2007; Schmiedek et al., 2009). For backward recall, the relative serial positions of the memory items must be updated at the point of recall, and for n-back the serial position of items previously encoded must be updated as new items are continuously presented (Redick & Lindsey, 2013). The reordering processes and the role of (re)binding items to the appropriate temporal context may be similar across paradigms (Oberauer, 2005; Redick & Lindsey, 2013; Szmalec et al., 2011). An alternative possibility is that individual differences in attentional control underlie performance on both the n-back and backward recall tasks, consistent with theories proposing that attentional control – the processes that enable us to selectively focus on task-relevant information in the presence of internal and external distractions – are important for many cognitive abilities including working memory (e.g., Engle, 2002; Engle & Kane, 2004; Kane & Engle, 2002).

Despite some similarities, these tasks differ in important ways, and the importance of task-specific processes may explain why two separate paradigm-specific latent constructs best captured the data. That is, the paradigms may require different sequences of cognitive processes to be co-ordinated for task execution (e.g., Byrne et al., 2020; Gathercole et al., 2019). The tasks can be distinguished in terms of retrieval demands: backward recall involves explicit serial recall and retrieval is guided by self-generated cues (Kane et al., 2007), whereas n-back requires recognition and additional noise might be introduced by familiarity-based responding (Oberauer, 2005). The updating requirements also differ. For n-back, the full sequence must be refreshed as items are added and dropped; for backward recall the whole sequence must be maintained and transformed at the point of recall.

There was evidence for domain-specificity within the backward recall construct, but not in n-back: modification indices suggested that modelling residual covariance between the two verbal backward recall tasks, BDR and BLR, would provide a better account of the data, but the same was not true for the n-back tasks. Adding the residual covariance between the two verbal backward recall tasks accounted for the domain-specificity in backward recall and strengthened the relationship between the backward recall and n-back constructs. This suggests that the association between the n-back and backward recall constructs is domain-general. These data also indicate the mechanisms supporting n-back may be more domain-general and less dependent on stimulus material than backward recall (Jaeggi, Buschkuehl, et al., 2010) and/or that subvocal rehearsal is a less optimal strategy for n-back than for backward recall. The common variance between the two backward recall tasks using verbalisable materials, BLR and BDR, might be linked due to common maintenance processes. Both tasks may involve repeated cycles of forward verbal serial recall of diminishing lengths, peeling off the final item each time (Anders & Lillyquist, 1971; Gathercole et al., 2019; Thomas et al., 2003). The encoding and retrieval processes required from verbal short-term memory for verbal serial recall are also the same for digits and letters (Norris et al., 2019; Page & Norris, 1998): the system stores verbal material in phonological rather than semantic form (e.g., Salamé & Baddeley, 1982). In n-back there is less time for subvocal rehearsal as *n* increases, and the continuously updating nature of the task might make rehearsal a sub-optimal strategy. This means that verbalising may be a less important maintenance strategy in n-back, making performance more similar across verbal and spatial domains.

Exploratory analyses conducted to investigate the relationship between the backward recall and n-back factors and fluid reasoning revealed that a model with separate WM and reasoning constructs was preferred to a single-factor model encompassing both WM and reasoning. This supports the idea that WM and G*f* are highly related but dissociable constructs (Kovacs & Conway, 2016), and is consistent with many previous individual differences studies showing strong associations between WM capacity and general fluid reasoning (Chuderski, 2013; Engle & Kane, 2004; Engle, Laughlin, et al., 1999; Kane et al., 2004; Kyllonen & Christal, 1990; Schmiedek et al., 2009, 2014). While we cannot tease apart the mechanisms explaining these strong associations from the current data, candidate theories include a shared reliance of both WM and fluid intelligence on short-term memory (Colom, Rubio, Shih, & Santacreu, 2006; Colom *et al*., 2008) or the ability to control attention (Engle, 2018; Kane et al., 2007; Shipstead *et al*., 2016), or a role for WM processes in supporting performance on fluid reasoning tasks (Oberauer et al., 2007).

The two WM factors did not differentially predict fluid reasoning: a model in which the paths between each WM factor and fluid reasoning were constrained to be equal provided as good a fit to the data as a model in which they were allowed to vary freely. This suggests the data can be explained equally as well by assuming the links between each WM factor and reasoning are equal: there is no significant difference in the ways in which n-back and backward recall predict reasoning. This is inconsistent with previous results showing that different WM paradigms (visual array and complex span) predict unique variance in fluid reasoning (Shipstead et al., 2012). This inconsistency could reflect differences in the analytic approaches used across studies. Shipstead et al. (2012) used stepwise regression to isolate the unique contribution of each paradigm to reasoning, while the current study showed no differences in model fit between a model that assumed the contributions of both tasks to reasoning were equal and one that assumed they differed. Another difference is that the paradigms used by Shipstead et al. (2012) differed more substantially in terms of task demands. While visual array captured differences in the scope of attention, complex span captured differences in attentional control (Shipstead et al., 2012). Arguably the two tasks used in the current study – backward recall and n-back – are more similar to one another as both capture individual differences in attentional control.

Bifactor models, conducted to explore whether the common variance across all WM tasks was a stronger predictor of reasoning than the variance specific to either a residual backward recall or n-back factor, revealed that common WM variance was consistently related to reasoning, but the paradigm-specific residual variances varied depending on the model. While the backward recall variance predicted reasoning equally as strongly as the common WM variance factor, the variance specific to n-back was significantly less strongly related to reasoning than the common WM variance factor. Findings for the n-back residual factor support the domain-general view of WM (e.g., Kane et al., 2004), which emphasizes that the shared variance among WM paradigms should be a stronger predictor of complex cognitive abilities. The backward recall outcome, however, suggests the processes specific to backward recall that do not overlap with n-back, maybe the explicit resequencing of information (Thomas et al., 2003; Gathercole *et al*., 2019), are as important for reasoning as the overlapping domain-general attention control processes associated with both backward recall and n-back (e.g., Engle & Kane, 2004; Engle, Kane, et al., 1999). Together with the modelling of the WM tasks, which revealed the individual tasks were better represented as paradigm-specific factors corresponding to backward recall and n-back than a single WM factor, these data further cement the distinctiveness of n-back and backward recall. They also add to the debate around whether WM and fluid reasoning are isomorphic constructs (e.g., Duncan et al., 2000; Kyllonen & Christal, 1990). The WM and fluid reasoning associations were not even identical across WM paradigms, and the residual variance in fluid reasoning after accounting for that predicted jointly by the two WM constructs was significantly different to zero. Together, these findings support the idea that WM and fluid reasoning are distinct constructs (e.g., Kane et al., 2004; Oberauer et al., 2005; Schmiedek et al., 2009, 2014).

There is a debate concerning whether BDR is a measure of WM (Alloway et al., 2006) or a measure of short-term memory that draws on the strategic use of visual imagery (Rosen & Engle, 1997; St Clair-Thompson, 2010; St Clair-Thompson & Allen, 2013). The shared variance between the backward recall tasks and both the n-back and fluid reasoning constructs, and the fact that the backward recall factor was an equally strong predictor of reasoning as variance common to all the WM tasks, suggests backward recall shares common variance with other measures of higher-order complex cognition, indicating it may tap into more than just short-term memory.

### Limitations and future directions

Major strengths of the current study are the sample size, systematic manipulation of WM task features within and across paradigms, and the pre-registration. The protocol and analysis plan were preregistered via the Open Science Framework. Preregistering the models we planned to test *a priori* has enabled us to be clear about what our data do and do not confirm, a central scientific principle of latent variable methods. Open science practices like these should be the norm for all confirmatory factor analytic studies. It is important to note that preregistration does not undervalue exploratory research, but instead encourages researchers to clearly distinguish between planned analyses that are confirmatory, and those that are exploratory as we have exemplified in this paper. We note that we have made our data and code freely available and encourage readers to engage with our data to move the conversation further (e.g., by testing hierarchical or network models).

There were several limitations to the current study. First, ascending list-lengths with discontinue rules were used for the WM tasks. While this staircase procedure is an efficient way to capture overall WM capacity limits, other administration and scoring procedures (e.g., participants complete fixed list lengths for all levels of difficulty) would have allowed for partial credit scoring, and captured more variability in performance (e.g., Conway et al., 2005; St Clair-Thompson & Sykes, 2010; Unsworth & Engle, 2007). Second, stimuli were presented randomly in the n-back tasks, with no control over presentation of lures. Future work could adopt a parallel approach to this study but include near-n lures to minimise the contributions of familiarity, and maximise contributions of active WM processes, to the tasks (e.g., Szmalec et al., 2011). Third, the exploratory analyses relied on data from one fluid reasoning task. Ideally, we would fit our fluid reasoning latent variable based on multiple subtest scores, but these were not available. Instead, we used a model with two indicators reflecting the sums of the odd and even trials. This yielded (very) high factor loadings (> .95) for both indicators and empirical identification in the overall model. For these reasons, we think it unlikely our results would be meaningfully different with alternative specifications of the fluid reasoning latent variable. Finally, factor analytic approaches provide only one way to determine task overlap, and the patterns of association can be affected by external variables such as differences in the difficulty of the tasks. A fruitful avenue for future work to test the current findings further could involve developing formal measurement models for the different tasks, and evaluating how far the parameters of these models show conceptual overlap (e.g., Frischkorn et al., 2022; Oberauer, 2016).

### Summary

To conclude, backward recall and n-back tasks tap into distinct paradigm-specific processes that are highly correlated with one another and with fluid reasoning, but not so strongly that they indicate isomorphic constructs. These findings provide some support for domain-general views of WM that acknowledge the possibility of task-specific variance (e.g., that associated with task-related processes and mechanisms), but they emphasise the distinctiveness between backward recall and n-back both in the individual task modelling, and in explorations of the associations between the residual variance specific to each paradigm (after controlling for common WM variance) and fluid reasoning.

## Open Scholarship

This article has earned the Center for Open Science badges for Open Data and Preregistered. The preregistration, data, and analysis code are openly accessible at https://osf.io/9qarp/.

## CRediT author statement

Elizabeth M. Byrne: Conceptualisation; Methodology; Project Administration; Software; Formal Analysis; Investigation; Data Curation; Writing – Original Draft; Visualisation; Funding acquisition. Rebecca A. Gilbert: Software; Data Curation; Writing – Review & Editing. Rogier A. Kievit: Methodology; Formal Analysis; Writing – Review & Editing. Joni Holmes: Conceptualisation; Methodology; Writing – Original Draft; Supervision.

## Data sharing and data accessibility

The preregistration, dataset, and analysis code are freely available on the Open Science Framework: https://osf.io/9qarp/.
