# Supplementary material for: Evidence for separate backward recall and n-back working memory factors: a large-scale latent variable analysis
Source: Memory. 2024 Aug 26;32(9):1182–98. doi: 10.1080/09658211.2024.2393388 (PMC11441403; doi:10.1080/09658211.2024.2393388)
Supplement: Supplemental Material [file PMEM_A_2393388_SM1111.doc]

SUPPLEMENTARY MATERIALS

*Supplementary model diagrams*

For the following model diagrams, latent factors are shown in ovals and squares represent observed variables. BDR = backward digit recall, BLR = backward letter recall, BSR = backward spatial recall, NBD = n-back with digits, NBL = n-back with letters, NBS = n-back with spatial locations, RR_even = relational reasoning with even items, and RR_odd = relational reasoning with odd items. All parameter estimates shown are fully standardized.

*Figure S1*. Model C: a two-factor paradigm-based model without residual covariance between BDR and BLR.

*Figure S2*. Model J: a three-factor paradigm-based model without residual covariance between BDR and BLR.

*Figure S3*. Model K: a single-factor general ability model without residual covariance between BDR and BLR.

*Figure S4*. Model I: a single-factor general ability model with residual covariance between the BDR and BLR variables, and between the two reasoning tasks.

*Additional information for the primary analyses*

Comparisons of original models versus the same models with residual covariance between BDR and BLR using the χ^2^ difference test revealed that the revised single-factor WM model (Model E) was statistically superior to the single-factor model without the modification (Model A), ∆ χ^2^ = 377.66, ∆ *df* = 1, *p* < .001, the revised domain model (Model F) was significantly better than the original domain model (B), ∆ χ^2^ = 357.63, ∆ *df* = 1, *p* < .001, and that the modified two-factor paradigm model was a statistically better fit than the same model without the residual covariance (Model B), ∆ χ^2^ = 17.587, ∆ *df* = 1, *p* < .001. The fit indices of these models are summarized in Tables 3 and 4 (main text), for the original and revised models, respectively.

*Additional information for the exploratory analyses*

A χ^2^ difference test showed a superior fit for the paradigm/reasoning model with residual covariance between BDR and BLR (Model H) compared to the same model without the residual covariance (Model J), ∆ χ^2^ = 67.627, ∆ *df* = 1, *p* < .001, and for the single-factor general ability model (Model I) compared to the same single-factor model without the residual covariance (Model J), ∆ χ^2^ = 196.310, ∆ *df* = 2, *p* < .001. Fit indices of the models included in the exploratory analyses with and without the residual covariance are summarised in Tables 5 (main text) and S1 (below), respectively.

*Supplementary tables*

*Table S1*. Fit statistics for models J and K included in the exploratory analyses, without residual covariance between BDR and BLR

| *Model* | | χ² | *df* | *YB* | *RMSEA* | *CFI* | *AIC* |
| --- | --- | --- | --- | --- | --- | --- | --- |
| (J) | Three-factor paradigm & reasoning | 63.574 | 17 | .980 | .062 [.046, .079] | .976 | 22789 |
| (K) | Single-factor general ability | 364.420 | 20 | 1.074 | .157 [.143, .170] | .819 | 23112 |
| *Note*. For root mean errors of approximation (RMSEAs), 90% confidence intervals are given. CFI = comparative fit index; AIC = Akaike information criterion. The χ² reported is the Yuan-Bentler scaled χ², with the scaling factor reported as YB. | | | | | | | |

*Table S2*. Fit statistics for the SEM models predicting reasoning by the working memory factors in the exploratory analyses. Statistics for the freely estimated and equality constrained models are shown.

| *Model* | χ² | *df* | *YB* | *RMSEA* | *CFI* | *AIC* |
| --- | --- | --- | --- | --- | --- | --- |
| Freely estimated model (Model L) | **23.576** | 16 | 1.006 | .026 [.000, .047] | .996 | 22752 |
| Constrained model | 27.677 | 17 | 1.017 | .030 [.003, .049] | .995 | 22754 |
| *Note*. **Bold** text denotes a non-significant χ² value. For root mean errors of approximation (RMSEAs), 90% confidence intervals are given. CFI = comparative fit index; AIC = Akaike information criterion. The χ² reported is the Yuan Bentler scaled χ², with the scaling factor reported as YB. | | | | | | |

*Table S3*. Fit statistics for the SEM models predicting reasoning by the working memory factors (without the residual covariance between the BLR and BDR tasks) in the exploratory analyses. Statistics for the freely estimated and equality constrained models are shown.

| *Model* | χ² | *df* | *YB* | *RMSEA* | *CFI* | *AIC* |
| --- | --- | --- | --- | --- | --- | --- |
| Freely estimated model (Model M) | 63.574 | 17 | .980 | .062 [.046, .079] | .976 | 22789 |
| Constrained model | 66.239 | 18 | .997 | .062 [.046, .079 | .975 | 22790 |
| *Note*. For root mean errors of approximation (RMSEAs), 90% confidence intervals are given. CFI = comparative fit index; AIC = Akaike information criterion. The χ² reported is the Yuan Bentler scaled χ², with the scaling factor reported as YB. | | | | | | |

*Table S4*. Fit statistics for the bifactor model predicting reasoning by the common working memory variance and Backward recall factors in the exploratory analyses. Statistics for the freely estimated and equality constrained models are shown.

| *Model* | χ² | *df* | *YB* | *RMSEA* | *CFI* | *AIC* |
| --- | --- | --- | --- | --- | --- | --- |
| Freely estimated model (Model N) | **14.914** | 14 | .982 | .010 [.000, .039] | 1.00 | 22747 |
| Constrained model | **16.337** | 15 | .984 | .011 [.000, .038] | .999 | 22746 |
| *Note*. **Bold** text denotes a non-significant χ² value. For root mean errors of approximation (RMSEAs), 90% confidence intervals are given. CFI = comparative fit index; AIC = Akaike information criterion. The χ² reported is the Yuan Bentler scaled χ², with the scaling factor reported as YB. | | | | | | |

*Table S5*. Fit statistics for the bifactor model predicting reasoning by the common working memory variance and n-back factors in the exploratory analyses. Statistics for the freely estimated and equality constrained models are shown.

| *Model* | χ² | *df* | *YB* | *RMSEA* | *CFI* | *AIC* |
| --- | --- | --- | --- | --- | --- | --- |
| Freely estimated model (Model O) | **23.102** | 14 | .981 | .030 [.000, .052] | .995 | 22755 |
| Constrained model | 48.574 | 15 | .988 | .056 [.039, .075] | .982 | 22778 |
| *Note*. **Bold** text denotes a non-significant χ² value. For root mean errors of approximation (RMSEAs), 90% confidence intervals are given. CFI = comparative fit index; AIC = Akaike information criterion. The χ² reported is the Yuan Bentler scaled χ², with the scaling factor reported as YB. | | | | | | |
